# Crystallizable Fragment Glycoengineering for Therapeutic Antibodies Development

**DOI:** 10.3389/fimmu.2017.01554

**Published:** 2017-11-13

**Authors:** Wei Li, Zhongyu Zhu, Weizao Chen, Yang Feng, Dimiter S. Dimitrov

**Affiliations:** ^1^Protein Interactions Section, Cancer and Inflammation Program, Center for Cancer Research, National Cancer Institute, National Institutes of Health, Frederick, MD, United States

**Keywords:** monoclonal antibodies, crystallizable fragment glycosylation, homogenous glycoforms, effector function, crystallizable fragment glycoengineering, chemoenzymatic glycosylation remodeling, aglycosylated monoclonal antibodies, antibody–drug conjugate

## Abstract

Monoclonal antibody (mAb)-based therapeutics are the fastest growing class of human pharmaceuticals. They are typically IgG1 molecules with N-glycans attached to the N297 residue on crystallizable fragment (Fc). Different Fc glycoforms impact their effector function, pharmacokinetics, stability, aggregation, safety, and immunogenicity. Fc glycoforms affect mAbs effector functions including antibody-dependent cell-mediated cytotoxicity (ADCC) and complement-dependent cytotoxicity (CDC) by modulating the Fc–FcγRs and Fc–C1q interactions. While the terminal galactose enhances CDC activity, the fucose significantly decreases ADCC. Defucosylated immunoglobulin Gs (IgGs) are thus highly pursued as next-generation therapeutic mAbs with potent ADCC at reduced doses. A plethora of cell glycoengineering and chemoenzymatic glycoengineering strategies is emerging to produce IgGs with homogenous glycoforms especially without core fucose. The chemoenzymatic glycosylation remodeling also offers useful avenues for site-specific conjugations of small molecule drugs onto mAbs. Herein, we review the current progress of IgG-Fc glycoengineering. We begin with the discussion of the structures of IgG N-glycans and biosynthesis followed by reviewing the impact of IgG glycoforms on antibody effector functions and the current Fc glycoengineering strategies with emphasis on Fc defucosylation. Furthermore, we briefly discuss two novel therapeutic mAbs formats: aglycosylated mAbs and Fc glycan specific antibody–drug conjugates (ADCs). The advances in the understanding of Fc glycobiology and development of novel glycoengineering technologies have facilitated the generation of therapeutic mAbs with homogenous glycoforms and improved therapeutic efficacy.

## Introduction

**Figure 1:**
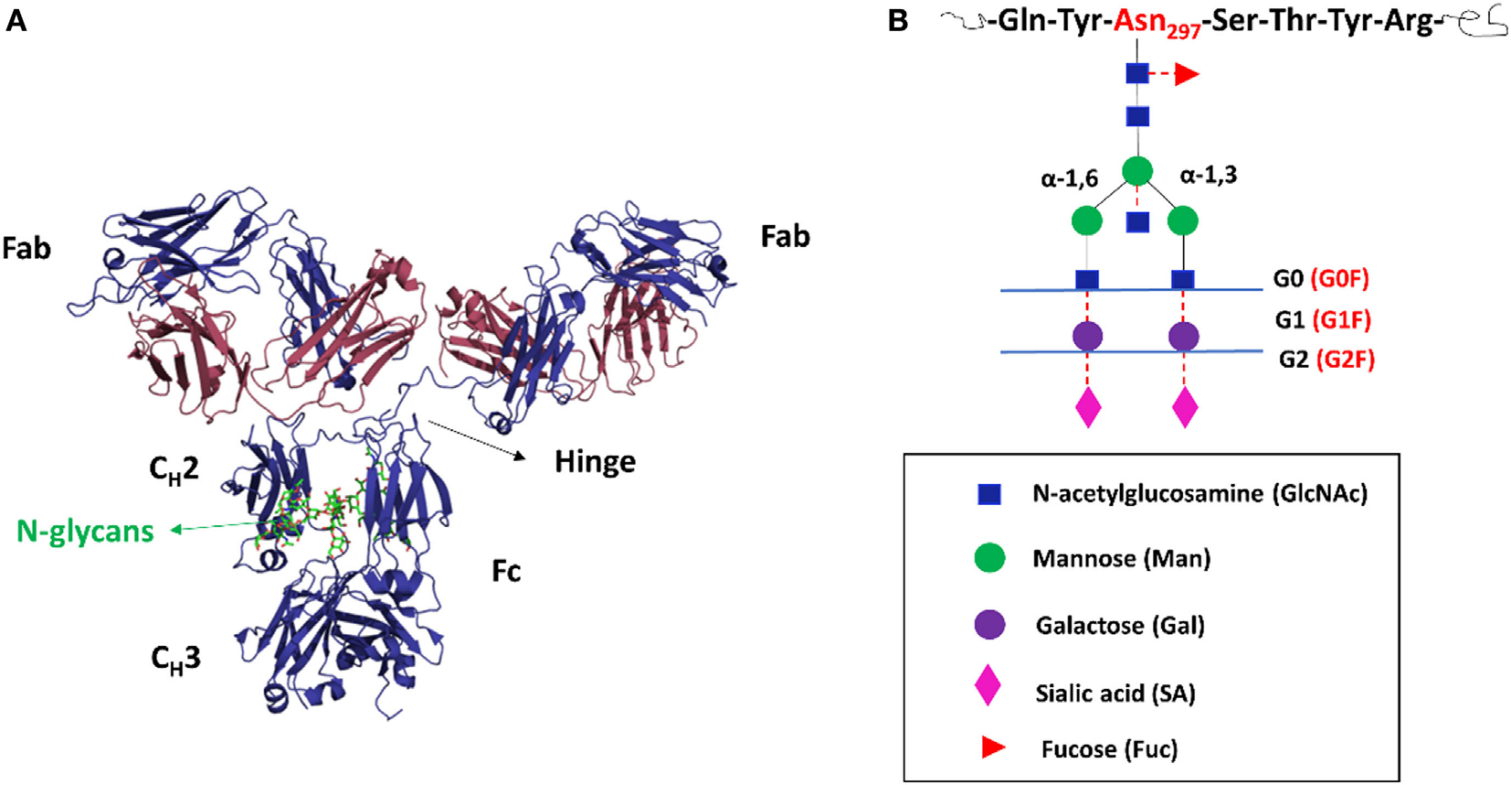
The structures of immunoglobulin G (IgG) and N-glycans. **(A)** Cartoon representations of a full-length IgG showing the functional domains. An IgG consists of two heavy chains (blue) and two light chains (red). The N-glycans are presented by the green color. Crystallizable fragment (Fc) is a dimer of C_H_2, C_H_3, and glycans. Antigen-binding fragment (Fab) is composed of variable heavy and light domains, as well as two constant domains (CH1 and CL). **(B)** The schematic structures of the possible biantennary oligosaccharides attached to human IgG-Fc at N297. The core heptasaccharide (G0) is linked in black lines; the outer arm sugar residues are attached to the core by the red dash line.

Monoclonal antibody (mAb)-based therapeutics have been the fastest growing class of human pharmaceuticals with applications in various clinical indications such as oncology, inflammatory diseases, organ transplantation, and bacteria and virus infection (1). Currently, more than 60 mAbs and derivatives are approved in USA and Europe for human use with some of them being blockbusters in the biopharmaceutical markets (2, 3). Under the vigorous engine of modern translational biotechnology, mAbs and derivatives are estimated to be >30% of the new licensed drugs (4). Most recombinant therapeutic mAbs are glycosylated immunoglobulin G (IgG) molecules with glycans attached to the amide nitrogen atom of asparagine 297 (N297) in the crystallizable fragment (Fc) region (Figure 1A) (5). It is well accepted that the N297-attached oligosaccharide is structurally integral to the IgG-Fc with multiple non-covalent interactions with the protein surface of the C_H_2 domain (6). The extensive carbohydrate–polypeptide interactions as well as carbohydrate–carbohydrate interactions modulate the conformations of the IgG molecules, which would ultimately impact the biological functions of mAbs (7).

During the last several decades, substantial knowledge has been acquired regarding the impact of Fc glycosylation on mAbs efficacy, pharmacokinetics (PK), stability, aggregation, safety, and immunogenicity (8–10). Many mAbs exhibit biological functions through immune effector functions including antibody-dependent cell-mediated cytotoxicity (ADCC), antibody-dependent cell-mediated phagocytosis (ADCP), and complement-dependent cytotoxicity (CDC) mediated by Fc–FcγR and Fc–C1q interactions (11). Alterations of glycoforms impact effector functions through modulating these Fc–ligand interactions (12–14). The effector functions of aglycosylated or deglycosylated IgGs are significantly dampened or eliminated due to the much lower binding to FcγRI or no binding to FcγRII and FcγRIII (15). Fc N-glycans impact stability of therapeutic antibodies in terms of shelf storage, thermal and chemical stability (such as pH and urea), aggregation propensity, susceptibility to proteolysis, *in vivo* clearance rate, and PK properties. The biophysical properties of therapeutic antibodies including the size, mass, charge, solubility, and colloidal stability are affected by N-glycans. Thus, different glycoforms could endow antibodies with distinct physicochemical and storage stabilities. Structurally, the glycans hold together with Fc C_H_2 domain with extensive non-covalent interactions, which not only protect the aggregation prone region (Phe241, Phe243, Pro244, Val262, Val264, Val303, and Val305) of C_H_2 from solvent exposure but also contribute to reduce the dynamics of C_H_2 and aid in C_H_2 folding (16, 17). These structural features could explain the decreased thermal, chemical stability, and increased aggregation propensity of aglycosylated IgGs compared with the glycosylated counterparts (16, 18, 19). In addition, the fact that the large complex type N-glycans with terminal galactose support an “open” Fc conformation compared with the “closed” Fc sustained by small glycans indicates N-glycans can also influence the folding of the Fc part (20). On the other hand, N-glycans impact the PK of IgG *via* modulating IgG sensitivity to serum protease cleavage. Due to the glycans protection, glycosylated IgGs are more resistant to trypsin, chymotrypsin, and pepsin than the aglycosylated IgGs (21). Glycoforms with distinct length, branching, and charge of sugar residues relate to the different susceptibilities of IgGs to proteolysis. While the terminal GlcNAc and sialic acid residues improve the resistance to proteolysis and hence enhance *in vivo* stability of IgG, terminal galactose residue confers higher sensitivity to proteases (22–24). The other way of selective clearance of glycosylated IgGs is executed by the C-type lectins mediated endocytosis. N-glycans with high mannose or terminated with GlcNAc could bind to the mannose receptors on macrophages/dendritic cells leading to the accelerated clearance of IgGs (25, 26). IgG with terminal galactose residue could be bound and cleared by the asialoglycoprotein receptor expressed in the hepatocytes (27). Besides, mAbs glycosylation also correlates with their immunogenicity and safety in humans. Therapeutic mAbs heterologously produced in CHO and murine myeloma cells (Sp2/0 and NS0 cells) possess non-natural sugars compared with human IgG, such as *N*-glycolylneuraminic acid (NGNA) residues and terminal α-1,3-linked galactose, which could induce potential immunogenicity in humans (28, 29).

Given its importance, glycosylation is considered a critical quality attribute for mAb therapeutics (30). The regulatory authorities require developers to keep glycoforms humanized and consistent with limited heterogeneity. Hence, developers need to strictly control the glycosylation profile during the development and production of mAbs. However, mAbs glycosylation is intrinsically heterogenous since glycans biosynthesis is not directly template driven. It is the product of sets of biochemical reactions involving a complex network of metabolic enzymes, which depends on the availability of sugar-nucleotide substrates, the enzyme distribution in the host cell, orchestrated process in endoplasmic reticulum (ER) and Golgi bodies and environmental factors (31, 32). Consequently, it is very challenging to achieve a highly homogenous glycoform independent of fermentation batches when expressing mAbs in eukaryotic cells. In the past decade, with significant advances in molecular and cell biology, protein and antibody engineering and gene editing, researchers have demonstrated individual glycoforms of antibodies could provide optimal efficacy for selected indications (Table 1) (33, 34). The pharmaceutical industry is increasingly pursuing the next-generation mAbs with tailored therapeutic effects. Herein, we review the current progress of mAbs Fc glycoengineering. We first present structures and biosynthesis of Fc N-glycans, followed by the discussion of impact of mAbs glycosylation on effector functions and the current glycoengineering strategies with emphasis on Fc defucosylation. Furthermore, we briefly discuss two novel therapeutic mAbs formats involving Fc glycans: aglycosylated mAbs and N-glycans targeted site-specific antibody–drug conjugates (ADCs).

**Table 1:** Selected glycosylation engineering of therapeutic antibodies for targeted diseases.

mAbs name	Target	Indication	Glycol modification	Development status	Reference
Otelixizumab	CD3	Type I diabetes, rheumatoid arthritis	Aglycosylated	Phase I (completed)	(35)
MTRX-1011A	CD4	Rheumatoid arthritis, cutaneous lupus	Aglycosylated	Phase I (completed)	(36)
Mogamulizumab	CCR4	ATLL, CTCL	Afucosylated	Approved	(37, 38)
MDX-1342	CD19	Relapsed or refractory CLL	Afucosylated	Phase I	(39)
Obinutuzumab	CD20	CLL, follicular lymphoma	Low fucose	Approved	(40)
DI-B4	CD19	CD19-positive indolent B-cell lymphoma	Low fucose	Phase I	(41)
RG7160	EGFR	EGFR-positive solid tumors	Bisected; non-fucosylated	Phase II	(42)
GTMAB2.5GEX	Mucin 1	*A-MUC1*-positive ovarian cancer	Glycooptimized	Phase II	(43)
Rituximab	CD20	CLL and NHL	Galactosylated	NA	(44)
Intravenous immunoglobulin	NA	Autoantibody-driven inflammation	Sialylated	NA	(13)

*CCR4, C–C chemokine receptor type 4; EGFR, epidermal growth factor receptor; ATLL, adult T-cell leukemia/lymphoma; CTCL, cutaneous T-cell lymphomas; CLL, chronic lymphocytic leukemia; NHL, non-Hodgkin’s lymphoma; NA, not applicable; mAb, monoclonal antibody*.

## IgG-Fc N-Glycans Structures and Biosynthesis

The IgG-Fc N-glycan is usually of limited size with no more than three antennae (33). Typically, oligosaccharides of normal human IgGs are biantennary complex structures with a core heptasaccharide and an outer arm of sugar residues (6) (Figure 1B). The core oligosaccharide (GlcNAc2Man3GlcNAc2, designated as G0) is composed of two inner GlcNAc, three mannoses, and two GlcNAc β-1,2 linked to α-3 and α-6 mannose forming two antennae (α-3 arm and α-6 arm). One major feature of IgG-Fc glycans is the microheterogeneity, which not only stems from the linkage of sugar isomers and glycosylation site occupancy but also results from the outer arm sugar addition depending on the expression system and glycosylation enzymatic machinery (31, 64). Such additions include fucose (Fuc, G0F), galactose (Gal, G1, and G2), bisecting GlcNAc (linked to the core GlcNAc-associated mannose, which is catalyzed by GlcNAc transferases III), and sialic acid including *N*-acetylneuraminic acid (NANA) or *N*-glycolylneuraminic acid (NGNA) residues (9). In addition, structural studies have shown that the two N-glycans from every heavy chain are asymmetrically oriented (65, 66), which further diversifies IgG-Fc glycoforms. Consequently, Fc N-glycans possess more than 400 glycoforms considering random pairing of two different heavy chain glycans (67). Human serum IgG-Fc glycans typically contain ~30% G0F, ~35% G1F, ~16% G2F, and ~15% bisecting GlcNAc (68). Low levels of sialylation are observed in human IgG-Fc glycans with monosialylated and disialylated glycoforms accounting for approximately 5–10 and 1%, respectively (69). Interestingly, mAbs produced in recombinant expression systems share similar N-glycans structures with IgGs from human serum (13). MAbs produced in CHO, NS0, and Sp2/0 cell lines have predominant glycoforms of G0F, G1F, G2F, a paucity of sialylated glycans and do not contain bisecting GlcNAc (34, 70).

**Figure 2:**
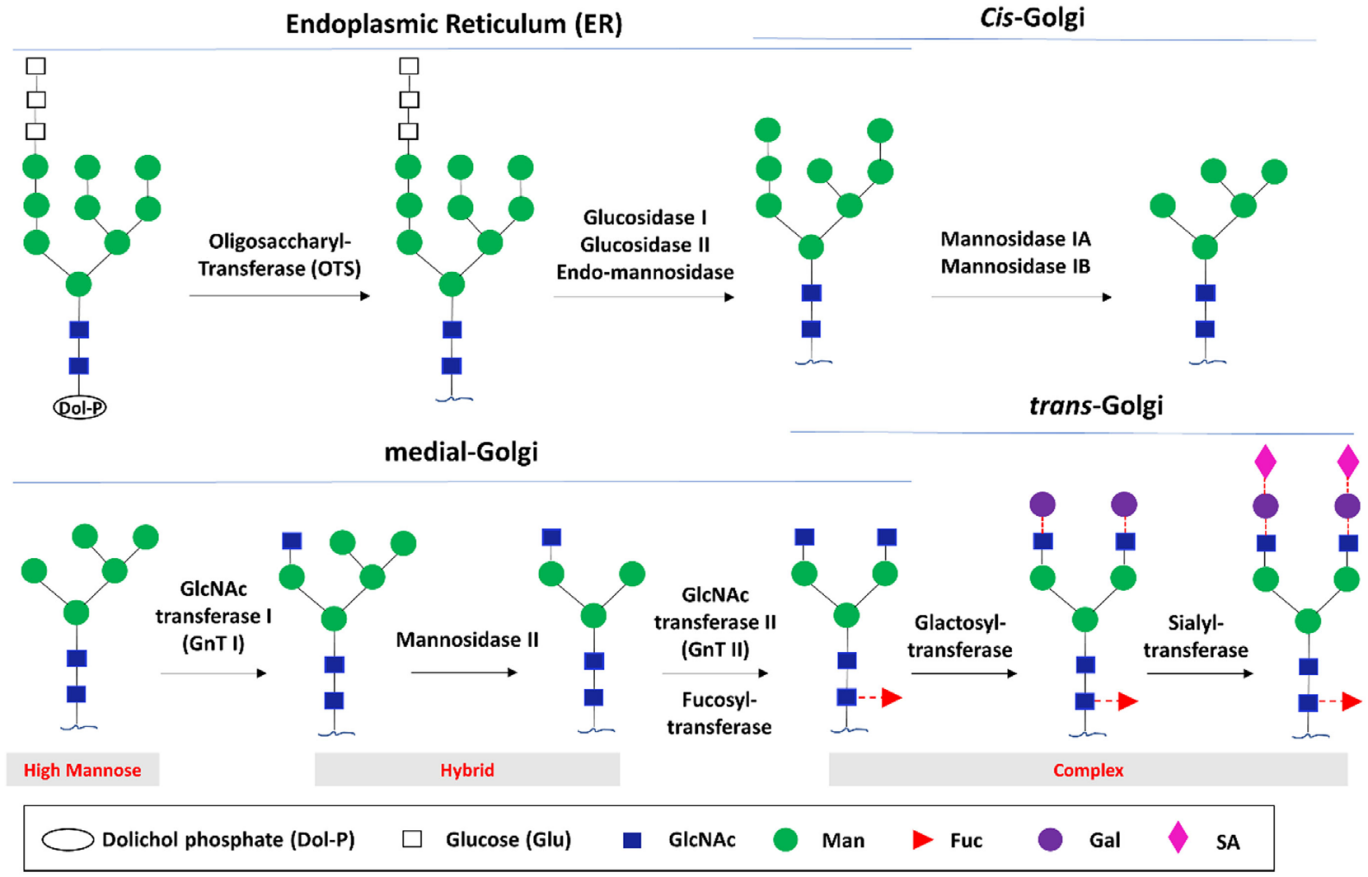
Glycan biosynthesis through the endoplasmic reticulum (ER) and Golgi glycosylation pathway. The biosynthesis begins with the processing of the initial high mannose N-glycan in the ER followed by transferring into the *cis*-Golgi to generate the core N-glycan substrate used for further diversification in the *trans*-Golgi. The potential glycoforms include the high mannose, hybrid, and complex structure.

Like other glycoproteins, glycosylation of IgG occurs through the conserved ER and Golgi glycosylation pathway (32). N-glycosylation begins with the addition of a pyrophosphate-dolichol precursor (Dol-P, Glc3Man9GlcNAc2) to the consensus N-glycosylation sequon (Asn–X–Ser/Thr, where X is any amino acid except Pro) of a nascent polypeptide by a transmembrane oligosaccharyltransferase (Figure 2) (71). Thereafter, the N-glycans are subjected to series of sequential modifications by sets of glycosidases and glycosyltransferases. In the lumen of the ER, polypeptide associated Glc3Man9GlcNAc2 is sequentially trimmed by glucosidases I and II and endo-mannosidase to yield Man8GlcNAc2 (72). This process is under protein folding quality control mediated by calnexin–calreticulin complex. In the *cis*-Golgi, the Man8GlcNAc2 is sequentially processed by two class I α-mannosidases that act specifically on α-1,2-Man residues to give rise to the core Man5GlcNAc2 glycan for further diversification in the medial and *trans*-Golgi, which include stepwise addition of the outer arm monosaccharide residues, catalyzed by GlcNAc transferases I, II, and III (GnT I, II, and III), fucosyltransferases, galactosyltransferases (GalT), and sialyltransferases (SiaT).

## Impact of Fc Glycosylation on IgG Effector Functions

**Figure 3:**
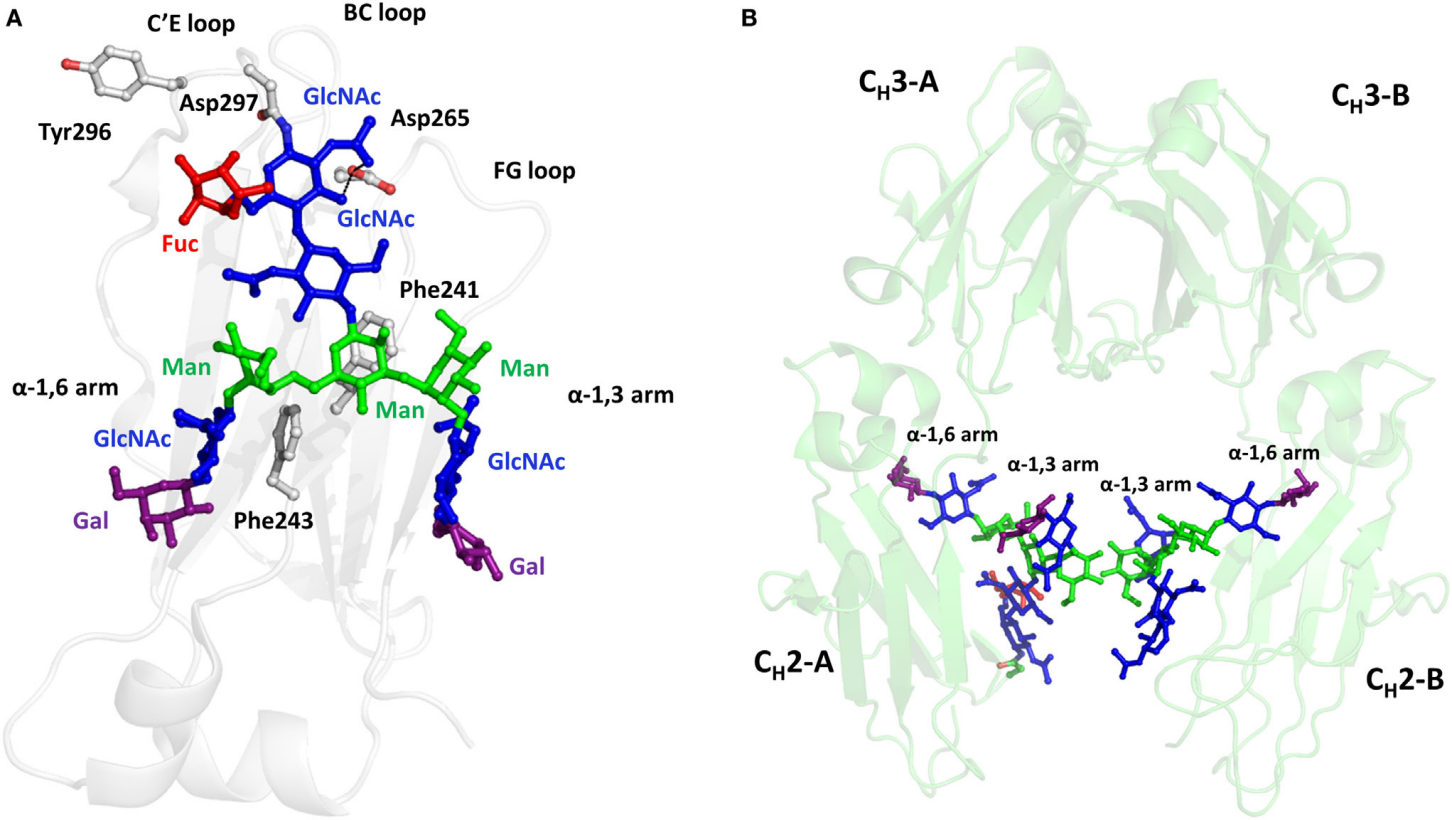
One X-ray crystal structure of N-glycan attached to N297 of crystallizable fragment (Fc) (PDB ID: 4CDH). **(A)** Cartoon representation of C_H_2 domain with N-glycans of biantennary complex structures. The sugar residues are represented as sticks and spheres models by PyMOL. Some non-covalent interactions between oligosaccharides and proteins are presented. **(B)** The structural orientations of N-glycans in Fc. The two glycans from each Fc pack against each other on the α-1,3 arms.

Crystallizable fragment glycoforms affect the effector function of antibodies by modulating the fine structure of Fc and thus altering Fc–ligands interactions. In recent years, structural insights into how antibody Fc glycoforms impact effector functions have been acquired by X-ray crystallography, nuclear magnetic resonance spectroscopy, and thermodynamics studies. Due to the stabilization effect of protein–sugar interaction, the core structures of GlcNAc2Man3 on the 1,3 arm and 1,6 arm are usually visible in the crystal structures. These two arms adopt distinct orientations—the 1,6 arm hangs over the hydrophobic face of C_H_2 domain while the 1,3 arm is orientated toward the internal space within the C_H_2 dimer (73). The oligosaccharide is well conserved and spans over 500 Å^2^ of the surface of each C_H_2 domains (20) (Figure 3A). Oligosaccharides make multiple hydrophobic and polar non-covalent interactions with the inner face of the C_H_2 domain (74). Impressive interactions include D265 hydrogen bonding to the inner GlcNAc and the α-1,6 arm forming strong CH-π packing with Phe241 and 243, which restricts the mobility of the glycans (75). Reciprocally, these intramolecular interactions restrain the C_H_2 conformation by stabilizing the C′E loop where the Asn297 locates, through which N-glycans pre-organize the ligands (FcγRs and C1q) binding interface on Fc (15). Besides, carbohydrate–carbohydrate interactions also contribute to maintain Fc conformation. The reciprocal mannoses from the two heavy chains make *sp*–*sp* contacts with each other (Figure 3B), which is necessary to establish a proper Fc conformation for ligand binding (14).

Multifaced impacts of terminal sugars on the antibody effector function have been elucidated. While high mannose, low fucose, and bisecting GlcNAc increase ADCC due to enhanced FcγRIIIa binding, terminal sialic acid decrease ADCC of IgG (14). For CDC, terminal galactose increases CDC by improving C1q binding, whereas terminal GlcNAc and sialic acid decrease CDC (12). Among these effects, reduction in fucose and terminal galactose, which improves ADCC and CDC, is highly desirable in antibody glycoengineering (76). Regulating α-2,6-linked terminal sialic acid is also an attractive strategy due to the anti-inflammatory role of these terminal sialic acid (77).

### Absence of Core Fucose Results in Improved ADCC Activity of IgG

**Figure 4:**
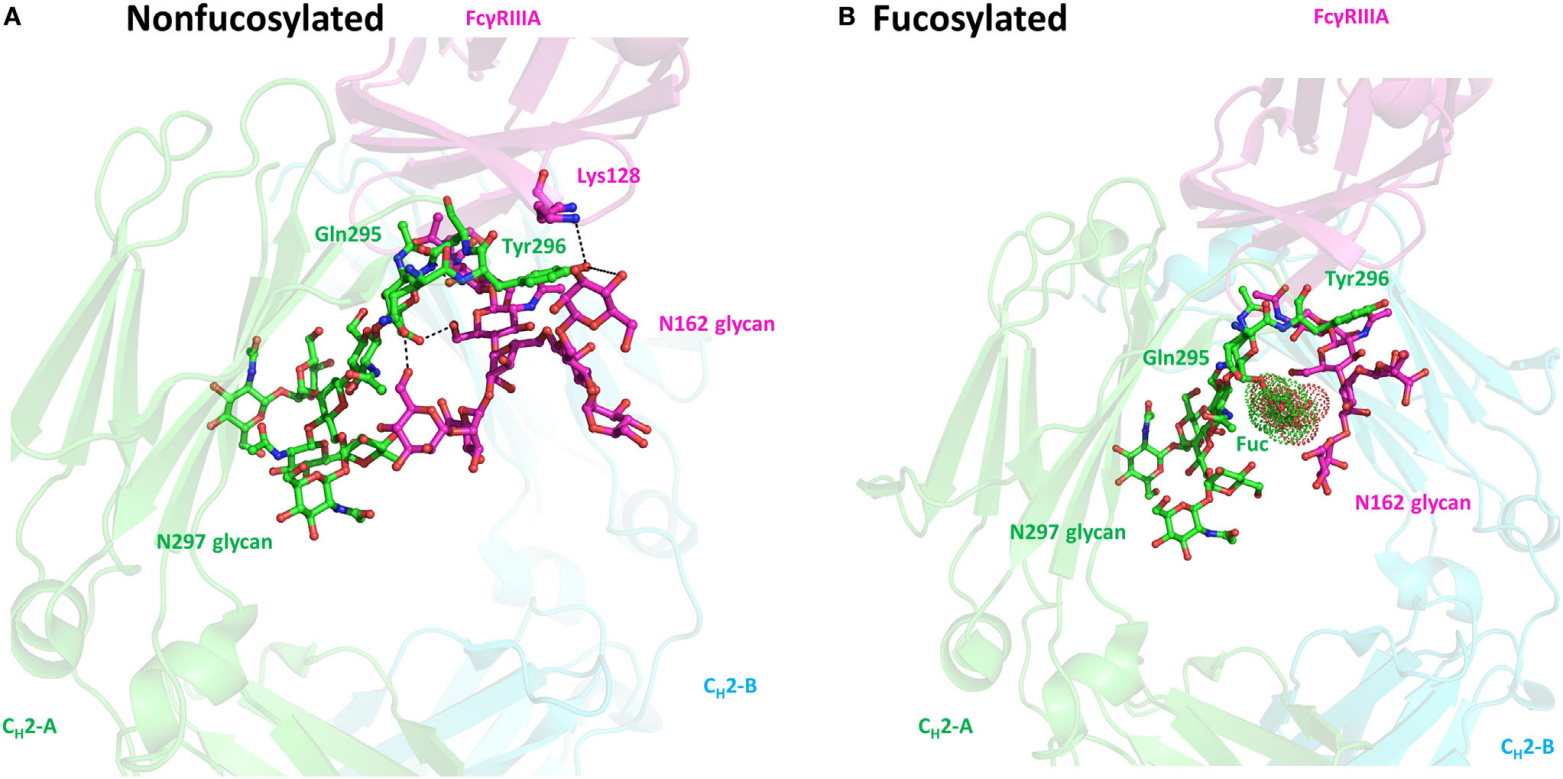
The crystal structures of non-fucosylated crystallizable fragment (Fc) (PDB ID: 3SGK) and fucosylated Fc (PDB ID: 3SGJ) complexed with FcγRIIIa with a high mannose glycan on N162. **(A)** Cartoon representation of non-fucosylated Fc–FcγRIIIa complex produced by PyMOL. The oligosaccharides and part of hydrogen bonding formation residues are shown in sphere and stick representation. The hydrogen bonds are depicted as black dash lines. **(B)** Cartoon representation of fucosylated Fc–FcγRIIIa complex. The core fucose locating at the interface of Fc N297 glycan and FcγRIIIa N162 glycans is highlighted with the dot representation.

Addition of a fucose to the innermost GlcNAc (the core fucose) is catalyzed by the α-1,6-fucosyltransferase in the medial-Golgi. More than 80% of the human IgG and >90% of the recombinant IgG produced by CHO cells contain the core fucose (13). However, the absence of core Fuc residue in the N-glycans significantly improves ADCC activity of IgG due to the substantially enhanced binding affinity to FcγRIIIa (31, 75). For example, afucosylated anti-HER2 IgG exhibits a ~100-fold greater ADCC effect compared with the fucosylated counterpart (51). The defucosylated antibody is also more potent than the fucosylated counterpart in the clinic (37, 78). The resolved complex crystal structures of Fc–FcγRIIIa have provided molecular rationales underlying the enhanced binding to FcγRIIIa for defucosylated IgGs. The crystal structure of sFcγRIIIa with high mannose-type glycans in Asn162 complexed with the defucosylated Fc show that the lower hinge regions of C_H_2 dimer dock onto the D2 domain of FcγRIIIa (79). Both the carbohydrate–carbohydrate and carbohydrate–protein interactions exist at the interface. The chitobiose core of Asn162 glycans hydrogen bond to the innermost GlcNAc of Fc. The 1,3-arm mannose of FcγRIIIa forms a hydrogen bond to the Gln295 of Fc. The branching β-mannose and Lys128 of FcγRIIIa make contacts with Tyr296 residue of Fc (Figure 4). However, these non-covalent interactions were unfavorable or disrupted due to the steric hindrances imposed by the presence of fucose in the fucosylated Fc. Besides, the conformation of Tyr296 is more constrained in the fucosylated Fc, which prevents Tyr296 from adapting a favorable conformation for binding to FcγRIIIa (80). Thus, the fucose moiety exerts allosterically inhibitory effects on the Fc–FcγRIIIa interaction, although it does not contact directly with FcγRIIIa. The enhanced binding affinity to FcγRIIIa endows defucosylated IgG several therapeutic merits. First, the high affinity could make the exogenous defucosylated IgG outcompete the endogenous serum IgG, thus avoiding the inhibitory effects of high concentration of serum IgGs on therapeutic IgG efficacies. For example, the inhibitory effect of endogenous IgG on ADCC was alleviated by defucosylated anti-CD20 antibodies (81, 82). Second, defucosylated antibodies have enhanced binding to the low affinity allotype of FcγRIIIa-158F and reduce the differences of ADCC efficacies of antibodies among the FcγRIIIa-158V and FcγRIIIa-158F allelic patients. Defucosylated IgGs have broader applications for all patients independent of the FcγRIIIa polymorphisms (83, 84). Finally, in addition to enhanced activation of FcγRIIIa-expressing killer cells (NK cells, monocytes, and macrophages) to mediate ADCC, the defucosylated antibody has also been reported to evoke ADCP effect through engaging FcγRIIIb on neutrophils, which in turn facilitates antigen presentation and recruitment of adaptive immunity, as evidenced by the defucosylated anti-CD20 IgG mediating upregulation of MHC class II molecules on neutrophil cell surface (85). Collectively, Fc fucosylation represents the most important influencer in modulating IgG effector function. Since ADCC is the main mode of action for mAbs in clinical oncology, defucosylated IgGs are highly desirable as the next-generation therapeutic antibodies. The high demand in defucosylated mAbs is driving the development of multiple glycoengineering strategies to produce low fucose antibodies (see below Table 2).

**Table 2:** Summary of the cell glycoengineering strategies to produce defucosylated antibodies.

Cell type			Glycoengineering modification	Company and technology platform	Antibody name	Targets	Development status	Reference
Non-mammalian cells	Yeasts		och or alg3 KO	NA GS4.0	Rituximab	Anti-CD20	NA	(45–47)
	Plants		RNAi of β-1,2-XylT and α-1,3-FucT	MAPP Biopharmaceutical	ZMAPP	Anti-Ebola	Phase 2/3	(48, 49)
Mammalian cells	YB2/0		Intrinsic low core fucose content YB2/0 (FUT8 low); Lec13 (GMD low)	LFB Biotechnologies EMABling Technology	Ublituximab; roledumab	Anti-CD20; anti-RhD	Phase 2/3	(50)
	CHO cells	Lec13 cells		Biowa	Hu4D5	Anti-HER2	NA	(51)
		WT CHO	Addition of sugar analog into culture medium (process engineering)	Seattle Genetics SEA Technology	SEA-CD40	Anti-CD40	Phase 1	(52)
		Genetically modified CHO	GMD KO	GMD knockout CHO/DG44	Rituximab	Anti-CD20	NA	(53)
			RMD overexpression	ProBioGen, GlymaxX Technology	Trastuzumab	Anti-HER2	NA	(54)
			GFT KO	CRISPR-Cas9 KO GFT CHO-F6	LSEVh-LS-F	Anti-HIV-1 Env	Pre-clinic	(55, 56)
			GnT III overpression	Roche GlycoMab Technology	Obinutuzumab	Anti-CD20	Approved	(57)
					RG7116	Anti-HER3	Phase 1	(58)
			RNAi of FUT8 and/or GMD	FG16	KM2160	Anti-CCR4	NA	(59, 60)
			FUT8 KO	Kyowa Hakko Kirin Potelligent Technology	Mogamulizumab	Anti-CCR4	Approved	(61)
					Benralizumab, ecromeximab, MEDI-551, BIW-8962, KHK2804, 2823, 2898, 4083	Anti-IL-5Rα, GD3, CD19, GM2, CD123, CD98	In clinical trials	(31)
				FUT8 knockout CHO/DG44	NA	Anti-CD20	NA	(62)
	293 FreeStyle cells		α-Mannosidase inhibitors kifunensine	NA	4Dm2m-F	Anti-HIV-1 Env	NA	(63)

*NA, not applied; RhD, rhesus D antigen; GFT, GDP-fucose transporter; GM2, ganglioside mono-sialic acid 2; GD3, ganglioside di-sialic acid 3; GMD, GDP-mannose 4,6-dehydratase; GnT III, GlcNAc transferase III*.

### High Galactose Enhances CDC Activity of IgG

**Figure 5:**
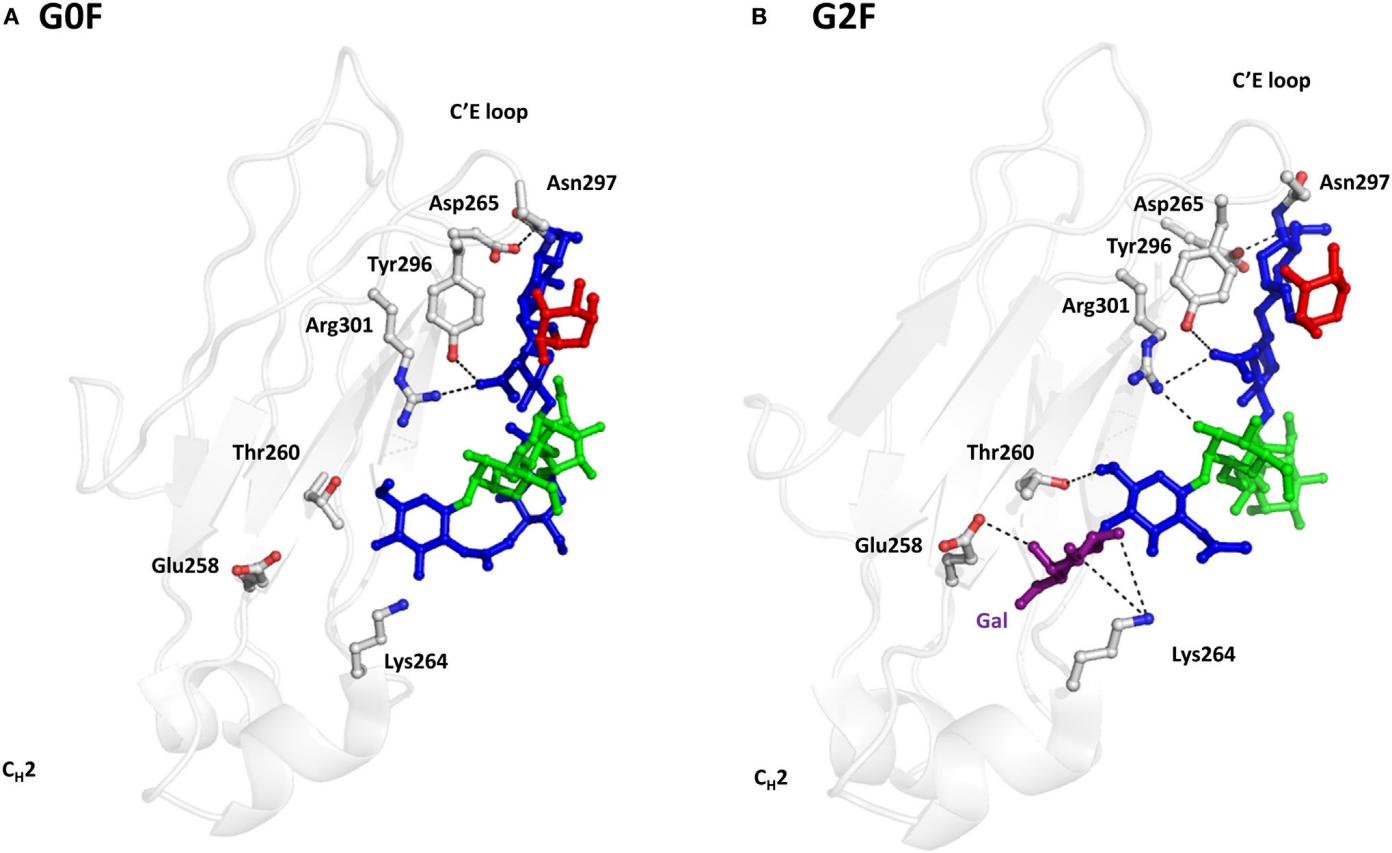
Representative crystal structures of IgG1 G0F, and G2F glycoforms. **(A)** The crystal structure of G0F. N-glycans and amino acids involving hydrogen bond formation are depicted by stick and sphere models. C_H_2 domain is represented as gray cartoon. The blue, green, and red colors represent GlcNAc, Man, and Fuc, respectively. Hydrogen bonds are drawn by black dash lines. **(B)** The crystal structure of G2F. The purple stick represents Gal, which engages several hydrogen bonds with nearby polar amino acids.

Both human serum IgG and recombinant IgG contain predominantly terminal galactose residues in their antennae (31). CHO cells-derived IgGs usually have lower levels of galactosylation compared with IgGs produced in mouse myeloma cells (32). Although the terminal galactose does not affect ADCC activity of IgG, it plays an important role in modulating the CDC activity (86). For example, the galactosylated rituximab exhibited higher CDC than the degalactosylated glycoform due to the higher affinity to C1q (44). Structurally, the extensive hydrophobic and hydrophilic interactions between terminal Gal residue and protein could impact the conformation of the C_H_2 domain, resulting in altered C1q binding (20). More hydrogen bonds between sugar residues and amino acids are found in the G2 glycoform compared with the G0 form of IgG1 (Figure 5) (14, 87, 88). Consequently, the stretch from residue 244 to 247 of C_H_2 domain is destabilized in the G0 glycoform, which was also supported by the comparative differential scanning microcalorimetry showing that G0 form associates with a lower enthalpy than the G2 form (89). These studies suggest that the non-covalent interactions between galactose and amino acid residues may account for the increased binding affinity between galactosylated Fc and C1q. Although the role of terminal galactose is not completely elucidated and in some cases the effect of terminal galactose has been reported to be antibody dependent, a proper control of galactosylation during manufacturing is still warranted.

### Impact of Terminal Sialylation on IgG Functions

The terminal sialic acid residue prolongs IgG half-life in the serum by shielding “galactose” residues from exposure to galactose-specific receptors in hepatocytes (32). On the other hand, sialylation may be undesirable because it renders IgGs more sensitive to protease compared with asialylated antibodies, probably due to the bulkier sialic acid leading to structural perturbations of C_H_2 domains (22, 90). The crystal structure has shown that the 1,6-arm sialic acid poses away from the protein-associated galactose residue and is entirely exposed to the solvent (91). The α-2,3-sialylation negatively impacts the canonical galactose-protein interactions and potentially destabilizes the C_H_2 domain (92). In addition, sialylation has negative effect on the ADCC activity of mAb (93), which may either stem from the decreased hinge flexibility upon sialylation causing reduced FcγRIIIa binding, or from the reduced bivalent antigen binding due to the lack of hinge flexibility (94). Antibody sialylation is desired due to its anti-inflammatory effects with potential applications in autoimmune and inflammatory diseases (95). The effect of sialylation was first discovered from human intravenous immunoglobulin but can also be recapitulated by the α-2,6-sialylation in recombinant IgG (96). Although not fully understood, this anti-inflammatory effect is triggered by the sialylated IgG interacting with the murine C-type lectin-like receptor-specific intracellular adhesion molecule-grabbing non-integrin R1 (SIGN-RI) on macrophage and dendritic cells (human ortholog, DCSIGN), which leads to increased FcγRIIb expression and expansion of Treg cell populations suppressing of inflammatory response (97, 98). Collectively, terminal sialic acid residues have both positive and negative effects on antibodies biological functions. It is necessary to strictly control sialylation in recombinant IgGs.

## IgG-Fc Glycoengineering

Since different glycoforms have distinct impacts on antibody effector function, it is necessary to control antibody glycoforms. With advanced knowledge of glycobiology, it is feasible to produce homogeneously glycosylated antibodies with tailored effector function. Strategies include host cell based glycoengineering involving manipulations of biosynthetic pathways and *in vitro* chemoenzymatic glycosylation remodeling.

### Cell Glycoengineering

Host cell glycoengineering has been highly pursued in recent years to produce recombinant IgG with desired glycoforms. As mentioned above, antibody glycoforms resulting from sets of enzymatic reactions pathways are a combined function of host cells, enzyme kinetical parameters, nucleotide sugar substrates, and the external factors. Following this lead, we classify cell glycoengineering strategies into the following four types.

#### Selection of Cell Type, Environmental Factors, and Cell Culture Conditions

Antibody glycosylation is largely influenced by the host cells from which they are manufactured. mAbs produced by CHO cells are somewhat under-sialylation due to the lack of α-2,6-sialyltransferase in these cells (31). Host cells with intrinsically low α-1,6 fucosyltransferase activity could be used to produce IgGs with low core fucose (99). For example, the rat hybridoma cell line YB2/0 with low FUT8 activity, a type of α-1,6 fucosyltransferase responsible for adding the core fucose, is used for the productions of defucosylated IgG (50). Another example is the Lec13 cell line, a derivative of CHO cells with deficiency in GDP-mannose 4,6-dehydratase (GMD) function leading to low fucosylation (51). On the other hand, the cell culture environment could be manipulated during the fermentation process to alter and optimize antibody glycoforms (process glycoengineering) (32). For example, addition of uridine, manganese chloride, and galactose could increase terminal Gal to enhance CDC activity of IgG (76, 100). Addition of UDP-GlcNAc and using serum-free culture increased sialylation of IgG1 (101). Addition of modified sugars such as 2-fluorofucose to the culture medium inhibits core fucose incorporation (102).

#### Using Enzyme Inhibitors to Intervene Host Biosynthesis Pathway

**Figure 6:**
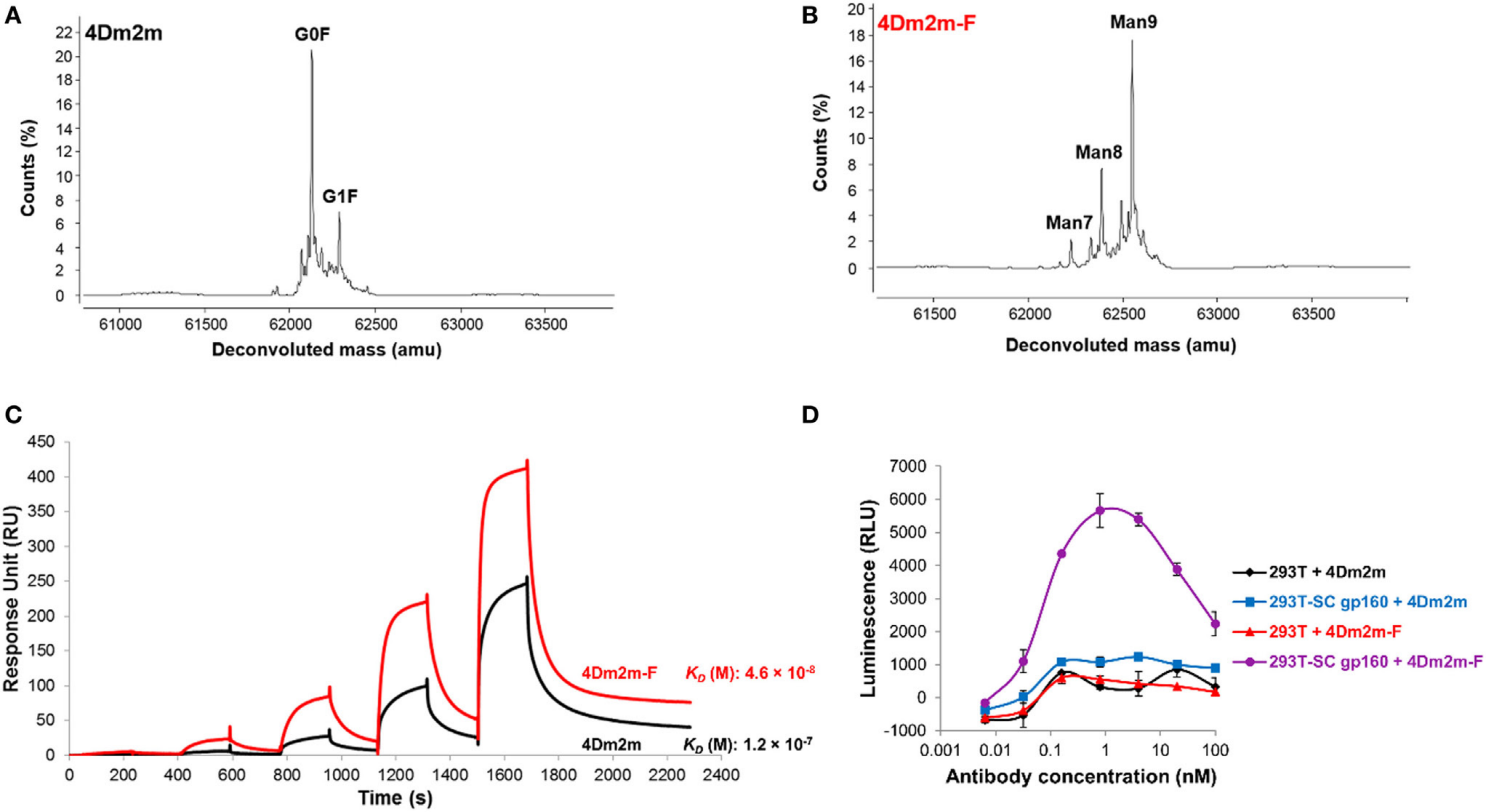
Use of the endoplasmic reticulum α-mannosidase inhibitors, kifunensine, to produce the high mannose glycoform with low fucose. **(A,B)** Deconvoluted mass spectra for heavy chains of 4Dm2m produced in the culture medium without **(A)** or with addition of kifunensine **(B)**. 4Dm2m and 4Dm2m-F were treated in buffer (7.5 M guanidine–HCl, 0.1 M Tris–HCl, and 1 mM EDTA) in the presence of 20 mM DTT and incubated at 70°C for 15 min. Mass spectrometry data were acquired on an Agilent 6520 Accurate-Mass Q-TOF LC/MS System. **(C)** Binding affinity to FcγRIIIa measured by surface plasmon resonance on a Biacore X100 (GE Healthcare) using a single-cycle approach. **(D)** Comparisons of antibody-dependent cell-mediated cytotoxicity (ADCC) activity of 4Dm2m and 4Dm2m-F by using the Promega ADCC reporter assay. Jurkat T cells engineered to express human FcγRIIIa and luciferase, through which ADCC signals were monitored.

Inhibitors able to modulate mAbs glycosylation have been reported. Antibody glycosylation is the result of multiple stepwise events. Enzyme inhibitors arresting mAbs in the intermediate glycoforms could prevent the additions of outer arm sugar residues including fucose (13). Such examples include the ER glucosidases I and II inhibitors, deoxynojirimycin and castanospermine producing Glc3Man9GlcNAc2 glycoform; the ER α-mannosidase inhibitors, deoxymannojirimycin and kifunensine producing the high mannose (Man9GlcNAc2) glycoform; and Golgi α-mannosidase II inhibitor swainsonine producing hybrid glycoforms such as GlcNAcMan5GlcNAc2Fuc (103). The authors’ group has used kifunensine to produce a defucosylated IgG-like bispecific and multivalent anti-HIV-1 molecule, 4Dm2m-F (63). 4Dm2m-F exhibits approximately threefolds higher binding affinity to FcγRIIIa than fucosylated 4Dm2m (Figure 6). The ADCC activity of 4Dm2m-F is also significantly improved based on the Promega ADCC reporter assay.

#### Genetic Modifications of the Host Biosynthesis Pathway

**Figure 7:**
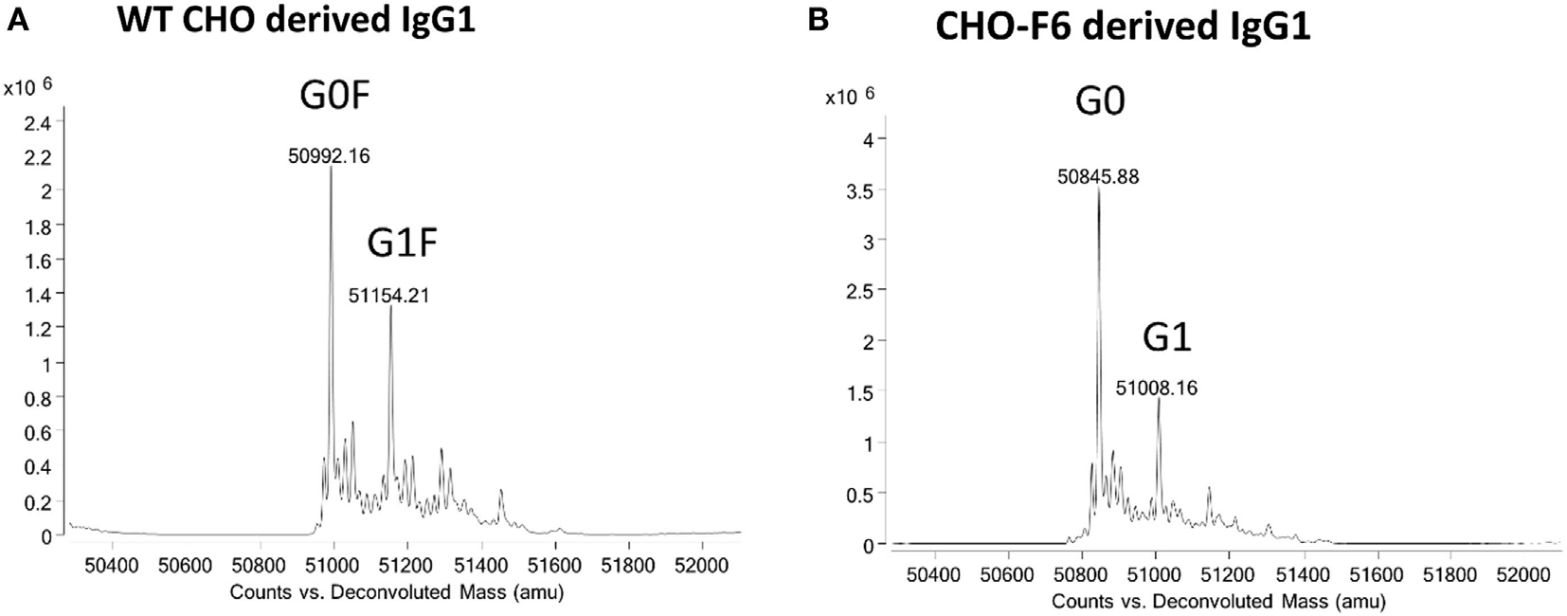
Knockout of GDP-fucose transporter gene in CHO cell line to generate CHO-F6 cell line for the production of afucosylated immunoglobulin Gs. **(A)** Deconvoluted mass spectra for heavy chain of m860 produced in the wide-type CHO cells (110). **(B)** Deconvoluted mass spectra for heavy chain of m860 produced in the CHO-F6 cell line. M860 and m860-F were treated in buffer (7.5 M guanidine–HCl, 0.1 M Tris–HCl, and 1 mM EDTA) in the presence of 20 mM DTT and incubated at 70°C for 15 min. Mass spectrometry data were acquired on an Agilent 6520 Accurate-Mass Q-TOF LC/MS System.

Antibody glycoforms can be altered by modulating host N-glycosylation pathway. The substrate availability can be changed by inactivation or overexpression of the corresponding nucleotide sugar transporters. For example, knockout of the mammalian *GMD* gene decreases the synthesis of the fucose donor, GDP-fucose, leading to production of defucosylated IgG (104). A similar method involves the overexpression of the GDP-6-deoxy-d-lyxo-4-hexulose reductase (RMD) (ProBioGen, GlymaxX technology) (105). In another example, co-transfection of cytidine mono-phosphate-sialic acid synthase, cytidine monophosphate-sialic acid transporter, and α-2,3-SiaT in CHO cell lines significantly has increased the intracellular CMP–SA level and improved the SA content of the recombinant protein (106). Recently, the gene editing technology is also used to engineer defucosylated antibodies. ZFNs and TALENs were used to inactivate GDP-fucose transporter (GFT) gene (Slc35c1) in CHO cells for production of fucose-free antibodies (107). Our group recently has used CRISPR-Cas9 to knockout GFT gene in CHO cell line (termed as CHO-F6) for the production of various afucosylated mAbs and Fc-fusion proteins (Figure 7) (55). Alternatively, the unwanted glycan pathways could be outcompeted by desired ones. For example, Roche’s GlycArt technology overexpresses β-1,4-GlcNAc transferase III (GnT III) to inhibit the downstream α-1,6-fucosyltransferase processing leading to the bisecting GlcNAc glycoforms rather than fucosylation (108). This technology has been used to produce the FDA approved anti-CD20 obinutuzumab (Gazyva^®^) (57). Co-overexpression of GnT III and α-mannosidase II leads to further lower fucose content by introducing non-fucosylated hybrid-type glycans (109). Another approach to control the desired or unwanted glycoforms is either genetically inactivating or increasing glycosyltransferases activity directly responsible for transferring of single monosaccharides to glycan structures. For sialylation, overexpression of α-2,3-sialyltransferase and β-1,4-galactosyltransferase elevates IgG sialylation and galactosylation (32). For fucosylation, genetically dampening FUT8 expression encoding α-1,6-fucosyltransferase significantly decreases or completely abolishes the terminal fucose (99). Small interfering RNA (siRNA) technique has been used to reduce α-1,6-fucosyltransferase activity for production of partially defucosylated IgGs (59). Furthermore, double siRNA knockdown of *FUT8* and *GMD* gene achieves completely afucosylated IgG (60). Alternatively, knockout of *FUT8* gene by the disruption of the genomic locus *via* homologous recombination could result in 100% afucosylated IgGs (62). One such afucosylation platform is the Potelligent Technology from Kyowa Hakko Kirin company utilizing *FUT8* KO CHO cell line to develop the anti-CCR4 mogamulizumab (Table 1, the first approved glycoengineered antibody) (61).

#### “Humanization” of N-Glycosylation Pathway of Non-Mammalian Cells

Some non-mammalian cell lines have been used to produce therapeutic antibodies due to the cost-effectiveness and/or decreased fucosylation (31). Glycoengineering of non-mammalian cells aims to humanize the immunogenic glycoforms by eliminating enzymes responsible for adding non-mammalian glycans and subsequently introducing the mammalian glycan processing enzymes. For example, knockout of the *och* or *alg3* genes in yeasts and knockout of plant-specific β-1,2-xylosyltransferase (β-1,2-XylT) and α-1,3-fucosyltransferase (α-1,3-FucT) genes achieve the elimination of the high mannose glycoforms and β1,2-xylose and core α1,3-fucose. The mammalian glycan processing enzymes such as mannosidases I and II, GnT I and II, β-1,4-GalT, and α-2,6-SiaT are then used to introduce human IgG-like glycoforms (13). Examples include the production of the rituximab in *Pichia pastoris* (45), the anti-HIV mAb 2G12 in *Nicotiana benthamiana* (111), and the anti-Ebola ZMAPP antibody cocktail (lack of core fucose) in plant (48).

### Chemoenzymatic Glycoengineering

Despite much progress in cell glycoengineering, it is still very challenging to produce IgGs with highly homogenous glycoforms in host cells. Consequently, the *in vitro* chemoenzymatic glycosylation remodeling provides an attractive alternative for the production of therapeutic mAbs with predefined and homogeneous glycoforms (34, 112). This method usually contains three steps: deglycosylation of IgG by an endo-β-*N*-acetylglucosaminidase (ENGase, such as endoglycosidase S, EndoS), simultaneously leaving the innermost GlcNAc at N297; preparation of oxazoline derivatives of customized N-linked glycan structures as sugars donors by chemical methods; transglycosylation of the glycan oxazoline donor to the innermost GlcNAc acceptor (113). The transglycosylation usually proceeds stereo-specifically under the catalysis of an ENGase, which was engineered to abolish the hydrolytic activity and improve substrate specificities (e.g., EndoS D233Q, EndoA N171A, EndoA E173Q, EndoMN175A, and EndoM N175Q) (114, 115). This chemoenzymatic approach has been successfully used to produce homogenous Fc glycoforms including non-fucosylated, fully sialylated and bisecting GlcNAc (116, 117). For example, rituximab was engineered from G0F, G1F, and G2F glycoforms to G2 and G2S2F glycoforms by EndoS-D233A and D233Q (114). Recently, the chemoenzymatic glycosylation remodeling was also elegantly used for site-specific conjugation of drugs onto antibodies (see below).

## Aglycosylated Full-Length IgG as a Novel Therapeutic Format

In recent years, aglycosylated full-length IgGs have gained substantial attentions due to their novel features (43, 118). Although the absence of N-glycans leads to the “closed” conformation of Fc and destabilization of C′E loop, the overall structures of aglycosylated IgG are similar to the glycosylated counterparts (119, 120). Thus, aglycosylated IgGs have almost identical antigen-binding affinity and pH-dependent FcRn binding and hence PK to glycosylated IgGs (121), which endows aglycosylated mAbs applications in the cases not requiring or avoiding undesired effector functions such as receptor blocking, targeted delivery, and anti-inflammation (122). Aglycosylated mAbs can be either produced in prokaryotic hosts (*E. coli*) or in eukaryotic hosts by introducing mutations at the Fc N297 or by the chemoenzymatic methods such as EndoS and PNGase-F enzyme treatment (120). Compared with the glycosylated IgG, aglycosylated IgG is devoid of glycan heterogeneity, hence significantly simplifies the biomanufacturing process leading to faster development timelines and lower developmental cost. Besides, aglycosylated IgG may be more susceptible to engineering due to the higher flexibility of the Fc conferred by the lack of N-glycans (118). Aglycosylated Fc could be engineered to restore or even improve its binding to FcγRs compared with glycosylated counterparts, which potentially extends the applications of aglycosylated IgGs into the cases effector functions are needed. For example, aglycosylated IgG-Fc could be engineered to bind to FcγRIIa and FcγRIIb comparably with glycosylated IgG-Fc by introducing double mutations S298G/T299A (123). More importantly, the higher flexibility renders the aglycosylated Fc being relatively easily engineered to exhibit unique FcγR specificity and novel effector functions. Jung et al. have used high-throughput library screening to develop an aglycosylated trastuzumab variant with five mutations (S298G/T299A/N390D/E382V/M428L) in Fc exhibiting >160-fold enhanced binding to FcγRIIa-R131 and 25-fold increased selectivity to FcγRIIa-R131 over FcRIIγb compared with the wide-type trastuzumab (124). Aglycosylated IgGs have established a new way for immunotherapy. Currently, several aglycosylated antibodies are under clinical investigation for efficacy and safety (120).

## Fc N-Glycan Specific ADC

Antibody–drug conjugates are IgGs conjugated with cytotoxic small molecules through chemical linkers. By specifically targeting cancer cells and selective delivery of highly cytotoxic drugs, ADCs fundamentally revolutionized the way of cancer immunotherapy and chemotherapy (125). Currently, four ADCs (Adcetris^®^, Kadcyla^®^, Besponsa^®^, and Mylotarg^®^) have been approved by FDA with more than 80 ADCs under clinical evaluations (126). The mode of action of ADCs involves antigen-mediated endocytosis, followed by the release of drugs by either lysosomal degradation or hydrolytic/proteolytic cleavage (127). The methods for conjugating the small molecule drugs onto IgG represent one of the key technologies in ADC development. The conventional conjugation approaches involve random addition of drugs onto Lys or reduced Cys residues by amide coupling and maleimide alkylation chemistry, which leads to highly heterogeneous mixtures with different drug–antibody ratios and inconsistent yield (128). This heterogeneity negatively impacts the *in vivo* efficacy, stability, PK of ADCs (129). Thus, site-specific conjugation methods are highly pursued (130). Conjugation through IgG-Fc N-glycans represents one of the most widely used site-specific conjugation methods (131). Glycosite-specific conjugation proceeds with the introduction of a chemically active moiety onto the Fc N-glycans followed by reacting with payloads carrying another chemically active group. In this method, native IgG with heterogeneous N-glycans is deglycosylated by a wild-type endoglycosidase followed by the transglycosylation of a chemical group capped homogenous N-glycan substrate. The transglycosylation is catalyzed by an endoglycosynthase (a mutant of endoglycosidase) that lacks hydrolytic activity but possesses transglycosylation activity (132). Subsequently, the drug payload can be conjugated by biocompatible chemical reactions such as click chemistry and oximation. Boons et al. have reported the utilization of a sialyltransferase to attach an azido-tagged sialic acid moiety onto the galactosylated IgG N-glycan and conjugate the payload, doxorubicin on the azido group *via* the “click chemistry” (133). van Geel et al. reported a different method to produce ADC, which involved the endoglycosidase-mediated deglycosylation to obtain the Fuc-α-1,6-GlcNAc disaccharide glycoform of IgG, followed by the addition of azido-capped UDP-galactose catalyzed by a mutant galactosyltransferase (134). Our group has exploited a galactosyltransferase mutant (β-1,4-Gal-T1-Y289L) to achieve glycosite-specific conjugation by transferring the keto-tagged or azido-tagged galactose onto the degalactosylated G0F glycoform of IgG. The final ADC products, m860-AF (Auristatin F) ADC and m276-PBD (pyrrolobenzodiazepine) ADC, were obtained through the keto oximation-mediated addition of AF and the “click chemistry”-mediated addition of PBD dimer onto m860 IgG1 and m276 IgG1, respectively (110, 135). The glycosite-specific conjugation strategies provide novel routes for the preparations of ADCs with better homogeneity and drug to antibody ratios.

## Conclusion and Perspectives

Unlike DNA and protein synthesis, antibody glycosylation synthesis is not directly template driven but is rather a result of networks of enzymatic reactions. Both host cells and the culture environment impact antibody glycosylation. Recombinant mAbs produced in host cells carry heterogenous Fc glycosylation, presumably with more than 400 possible glycoforms. Different glycoforms affect the *in vivo* efficacy, effector function, PK, stability, aggregation, safety, and immunogenicity of IgG. Among these, the impacts of Fc N-glycans on antibody effector function including ADCC and CDC are widely studied. IgG N-glycans affect their ADCC and CDC activity by altering Fc conformations and modulating the non-covalent interactions between oligosaccharides and C_H_2 domains. While the terminal Gal enhances CDC activity, the core fucose significantly inhibits ADCC by sterically hindering the interactions between IgG-Fc and FcγRIIIa. Thus, the regulatory authorities require developers to keep glycoforms of mAbs consistent with limited heterogeneity, which has driven the development of multiple cell glycoengineering strategies to produce mAbs with desired glycoforms, especially without fucose. Although progress has been made, it is still challenging to consistently produce fully homogenously glycosylated antibodies by glycoengineered cell lines. The chemoenzymatic glycosylation remodeling offers revolutionized avenues to IgG with homogenous glycoforms. However, most current chemoenzymatic glycoengineering is still under lab-scale explorations, which is very challenging to scale up for industrial development. The chemoenzymatic glycoengineering approaches also provide novel routes for the productions of ADCs. On the other hand, the glyco-heterogeneity of mAbs could be bypassed by aglycosylated full-length IgGs. However, it remains to be seen for the outcomes of the clinical trials of aglycosylated antibodies in terms of the *in vivo* stability and immunogenicity. In the future, “omics” technologies and systems biology modeling hold promises to aid the glycoengineering for developing next-generation mAbs with homogenous glycoforms and improved therapeutic efficacy.

## Author Contributions

DD conceived the topic; WL wrote the manuscript; ZZ, YF, and WC revised the manuscript.

## Conflict of Interest Statement

The authors declare that the research was conducted in the absence of any commercial or financial relationships that could be construed as a potential conflict of interest.
